# Presence of the *Eucalyptus* snout beetle in Ecuador and potential invasion risk in South America

**DOI:** 10.1002/ece3.10531

**Published:** 2023-09-19

**Authors:** Verónica Crespo‐Pérez, J. Angel Soto‐Centeno, C. Miguel Pinto, Ana Avilés, Washington Pruna, Claudia Terán, Álvaro Barragán

**Affiliations:** ^1^ Laboratorio de Entomología, Museo de Zoología QCAZ I, Escuela de Ciencias Biológicas Pontificia Universidad Católica del Ecuador Quito Ecuador; ^2^ Department of Earth and Environmental Sciences Rutgers University Newark New Jersey USA; ^3^ Department of Mammalogy American Museum of Natural History New York New York USA; ^4^ Charles Darwin Research Station Charles Darwin Foundation Puerto Ayora Ecuador; ^5^ Laboratorio de Biología Molecular, Herpetología, Escuela de Ciencias Biológicas Pontificia Universidad Católica del Ecuador Quito Ecuador

**Keywords:** ecological niche model, forest pest, *Gonipterus platensis*, invasive species

## Abstract

*Eucalyptus* snout beetles are a complex of at least eight cryptic species (Curculionidae: *Gonipterus scutellatus* complex), native to mainland Australia and Tasmania, that defoliate *Eucalyptus* trees and are considered important pests. Since the 19th century, three species of the complex have been introduced to other continents. Here, we document the presence of *Eucalyptus* snout beetles in Ecuador. We used DNA data for species identification and unambiguously demonstrated that the Ecuadorian specimens belong to the species *Gonipterus platensis*, which has low genetic diversity compared with other species in the complex. We analyzed *G. platensis*' potential distribution in South America with ecological niche models and found several areas of high to intermediate climatic suitability, even in countries where the pest has not been registered, like Peru and Bolivia. Accurate identification of species in the *G. scutellatus* complex and understanding of their potential distribution are essential tools for improved management and prevention tactics.

## Graphical abstract

We report the presence of the Eucalyptus snout beetle, *Gonipterus platensis*, in Ecuador. Ecological niche modeling revealed areas of climatic suitability in South America where this species is present or could invade in the future.

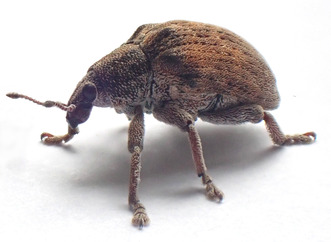

## Resumen

Los gorgojos del eucalipto son un complejo de al menos ocho especies crípticas (Curculionidae: complejo *Gonipterus scutellatus*), nativos de Australia continental y Tasmania, que defolian árboles de eucalipto y son considerados como plagas de importancia. Desde el siglo 19, tres especies de este complejo se han introducido a otros continentes. En este trabajo reportamos la presencia de gorgojos del eucalipto en Ecuador. Usamos datos genéticos para la identificación específica y demostramos claramente que los especímenes ecuatorianos pertenecen a la especie *Gonipterus platensis*, la cual tiene baja diversidad genética comparada con otras especies en el complejo. Analizamos la distribución potencial de *G. platensis* en América del Sur con modelos de nicho ecológico y encontramos varias áreas con idoneidad ambiental alta a intermedia, incluso en países donde esta especie no ha sido registrada, como Perú y Bolivia. La correcta identificación de las especies del complejo *Gonipterus scutellatus* y una mejor comprensión de su distribución potencial constituyen herramientas fundamentales para optimizar medidas de manejo y prevención.

## 1 INTRODUCTION

*Eucalyptus* trees (Myrtaceae) were initially introduced in South America in the early and mid‐19th century to meet increasing demands of wood, coal, and firewood in the region (FAO, 1981). Commercial plantations of *Eucalyptus* have rapidly expanded in the last three decades and together with pine trees, today they constitute the base of forestry development in many countries of South America (Estay, 2020). In Ecuador, several species of *Eucalyptus* (mostly *E. globulus*) were first introduced in 1865 largely for firewood and construction materials (FAO, 1981). They have also been extensively planted for reforestation and erosion control programs, making them very common and even dominant in many landscapes of the Ecuadorian Andes (Granda, 2006). Although *Eucalyptus* trees mainly occur in the inter‐Andean valleys of the country, a massive plantation program to produce wood chips for paper pulp was established in the early 2000 in the coastal province of Esmeraldas. Today, *E. globulus* is one of the most important tree species in the Ecuadorian forestry sector, which directly employs more than 230,000 people (ca. 5.5% of the economically active population) and contributes around 2%–3% to Ecuador's GDP (Grijalva et al., 2015).

*Eucalyptus* snout beetles are a complex of at least eight cryptic species (*Gonipterus scutellatus* complex) that feed on *Eucalyptus* leaves. These beetles are native to Eastern Australia and Tasmania. Species of the complex have been accidentally introduced into Western Australia (at an unknown date), New Zealand in 1890, Africa in 1916, South America in 1925, Europe in 1975 and North America in 1994 (Schröder et al., 2020). In South America, they have been introduced in Argentina in 1925, Uruguay in 1937, Brazil in 1955, Chile in 1998 (Estay, 2020; González et al., 2010; Marelli, 1927), and more recently, Colombia in 2016 (Madrigal‐Cardeño, 2019; Schröder et al., 2020). The morphological similarity between the species of the *G. scutellatus* complex has led to uncertainty and confusion regarding the identity of introduced populations (Mapondera et al., 2012; Schröder et al., 2020). According to the most recent taxonomy, two species of the complex are invasive in South America: *Gonipterus platensis*—the most widely distributed worldwide—in Argentina, Brazil, Chile, Colombia and Uruguay, and *G. pulverulentus*, with a limited distribution outside of Australia, only in eastern South America (Uruguay, Brazil and Argentina) (Mapondera et al., 2012). Although closely related and very similar, differences have been found in the coloration and markings of various life stages between the species of the *G. scutellatus* complex (Oliveira et al., 2022; Rosado‐Neto & Marques, 1996). Such differences could represent cues for their natural enemies and influence their level of success as biocontrollers (Schröder et al., 2020). Furthermore, results of previous studies suggest interspecific differences in environmental and host preferences of the beetles (Newete et al., 2011; Oliveira et al., 2022; Riquelme Virgala et al., 2018), which could guide when deciding on the species of *Eucalyptus* to be planted. Thus, correctly identifying newly introduced populations of *Eucalyptus* snout beetles is a first essential step toward effective management and control strategies.

In areas outside of their native range, *Eucalyptus* snout beetles cause severe damage to *Eucalyptus* trees, with both adults and larvae feeding on leaves and producing important economic losses (Mapondera et al., 2012). Damage includes crown defoliation, stag‐horned or witches' broom appearance, epicormic and stunted growth, reduced tree vigor, and loss of apical dominance (CABI, 2021; Lanfranco & Dungey, 2001), all of which make the trees more susceptible to attack by other organisms (Fiorentino & de Medina, 1991). Indeed, *Eucalyptus* snout beetle infestations were projected to produce between 20% and 85% losses in wood production over a 10‐year growth period (Reis et al., 2012). These impacts have raised repeated concern and motivated the study and development of different management tactics, including chemical control (Lanfranco & Dungey, 2001; Mally, 1924), silvicultural control (Tooke, 1955), biopesticides (Echeverri‐Molina & Santolamazza‐Carbone, 2010; Santolamazza‐Carbone & Fernandez De Ana‐Magan, 2004) and biological control (de Souza, 2016; González et al., 2010; Hanks et al., 2000; Reis et al., 2012). The egg parasitoid, *Anaphes nitens*, has been the most widely used bio controller of *Eucalyptus* snout beetles, with mostly good but sometimes incomplete success (Garcia et al., 2019; Hanks et al., 2000; Loch, 2008; Mapondera et al., 2012; Valente et al., 2017). Failure to successfully control beetle populations has been attributed, in part, to a climatic effect and to temporal asynchrony between host and parasitoid (Cordero Rivera et al., 1999; Reis et al., 2012), but also to the target species not being a preferred host for the parasitoid (Mapondera et al., 2012). Interestingly, *A. nitens* has been recently reported in Ecuador (Salazar‐Basurto et al., 2023), but given the above‐mentioned caveats, further studies about the ecology, life history, and host preference of the beetles and their parasitoids would certainly contribute to effective biological control strategies. Indeed, additional natural enemies of the beetles have been identified to date, including other egg parasitoids, like *A. tasmaniae*, and *A. inexpectatus* (Hymenoptera: Mymaridae), the larval parasitoid *Entedon magnificus* (Hymenoptera: Eulophidae), and even entomopathogenic fungi, which could be used alone or in combination in biological control programs (de Souza, 2016; Garcia et al., 2019; González et al., 2010; Gumovsky et al., 2015; Lobos Peirano, 2018; Valente et al., 2017). Finally, given that these beetles are listed as quarantine pests by several plant protection agencies (e.g., EPPO, CPCC, NAPPO) (CABI, 2021), and the long‐term presence and uses of this tree in forestry in South America, countries that export *Eucalyptus* products should strive to detect, adequately manage or prevent the introduction and spread of these insects.

Identifying potentially suitable environments where introduced species may thrive can help in mitigation and conservation planning efforts. Potential distributions of alien species into new geographic areas can be estimated with ecological niche models. These predictive models provide estimates of species' potentially occupied environmental niches based on the relationship between their occurrences and the environmental characteristics of the landscapes where they occur (Jiménez‐Valverde et al., 2011; Peterson et al., 2011; Soto‐Centeno & Steadman, 2015). Such procedures enable the construction of risk maps that identify areas suitable for invading or potentially invasive alien species and may aid in the implementation of successful biosecurity strategies (Pili et al., 2020).

**FIGURE 1:**
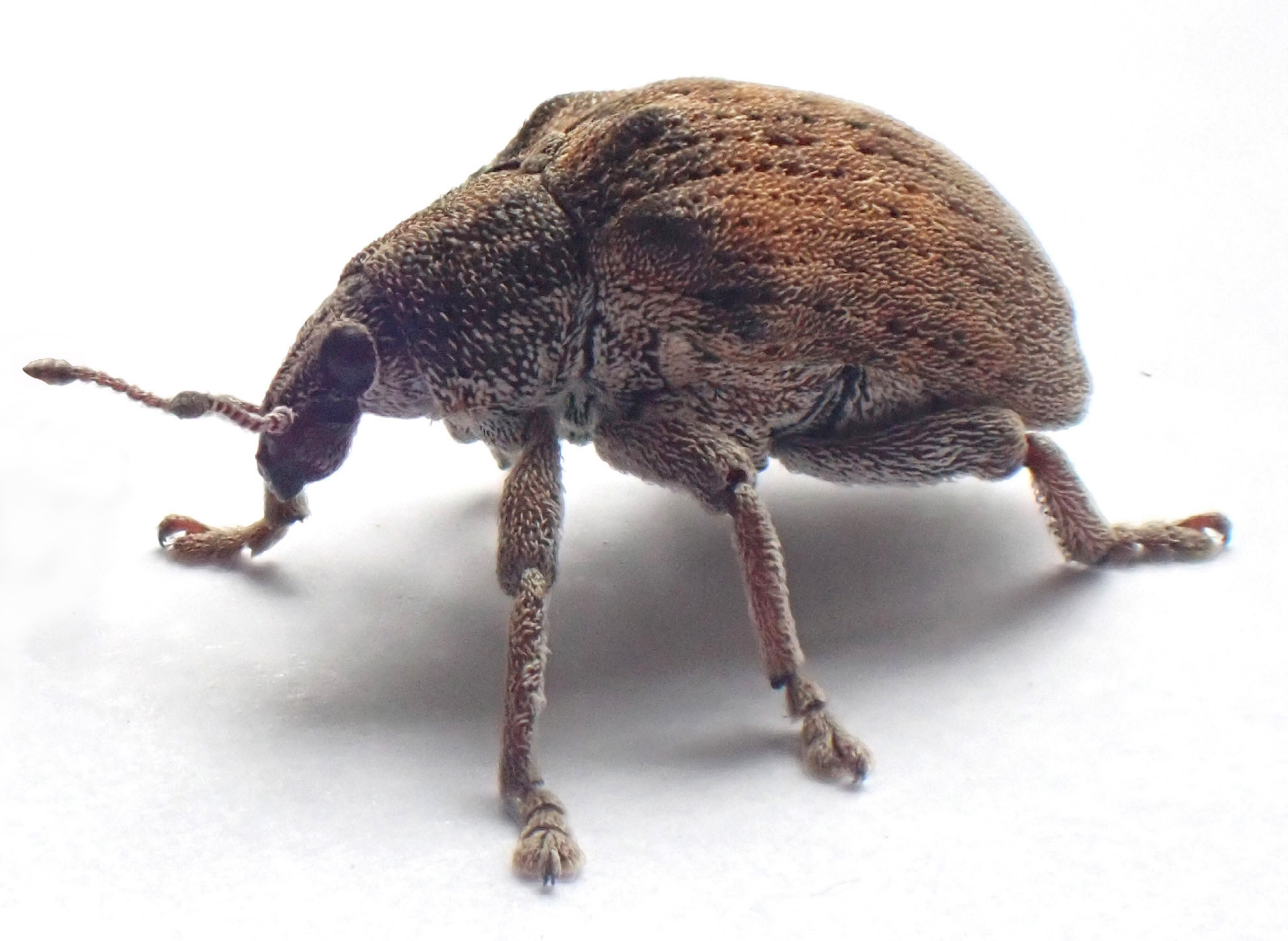
Adult of *Gonipterus platensis* collected by VC‐P and CMP in the city of Quito. Photograph by VC‐P with an Olympus Tough TG6 digital camera.

In this study, we report for the first time the presence of *Eucalyptus* snout beetles in the city of Quito, Ecuador (Figure 1). To determine to which species of the *G. scutellatus* complex these new records belong, we sequenced the COI gene of nine specimens collected in different sites in Quito and inferred a phylogeny using additional published sequences. After confirming their identity as *G. platensis*, we followed an ecological niche modeling approach to determine areas of suitable habitat in South America, with a focus on Ecuador and set a baseline for possible areas at risk of invasion.

## 2 METHODS

### 2.1 Specimen collection, DNA extraction, PCR, and sequencing

Specimens of the *Eucalyptus* snout beetle were collected by hand in various sites in the city of Quito. We removed three legs of the same side of nine individuals for molecular analyses. Voucher specimens were deposited in the invertebrate collection of the Zoology Museum (Quito Católica Zoología Invertebrados, QCAZ I) of Pontificia Universidad Católica del Ecuador (PUCE), Quito, Ecuador.

For DNA extraction, we left the legs in 500 μL of TE buffer for 24 h at 4°C. After rehydration, each softened leg was crushed with a pestle for 15 min, then 500 μL of CTAB‐PVP and 16 μL of Proteinase K were added to each sample and left shaking at 56°C for 24 h. We then added 650 μL of chloroform to each sample and mixed them to form an emulsion; subsequently, samples were centrifuged for 10 min at 13000 rpm. We removed the supernatant from every sample and saved each in different 1.5 μL tubes. Around 600 μL of 100% Isopropanol were added to every sample for DNA precipitation, and then centrifuged for 10 min at 13,000 rpm. The pellet was then washed with 70% ethanol and centrifuged again for 10 min at 13,000 rpm. The pellet was left to dry, and then resuspended in 25 μL of 0.1X TE Buffer. DNA concentration was quantified using a Nanodrop 1000 from Thermo Scientific.

We amplified two fragments of the mitochondrial gene COI with the following pairs of primers: the forward primer Jerry (5′‐CAA CAT TTA TTT TGA TTT TTT GG‐3′) and the reverse primer Pat (5′‐TCC AAT GCA CTA ATC TGC CAT ATT A‐3′) (Simon et al., 1994) and the forward primer dgLCO1490 (5′‐GGT CAA CAA ATC ATA AAG AYA TYG G‐3′) and the reverse primer dgHCO2198 (5′‐TAA ACT TCA GGG TGA CCA AAR AAY CA‐3′) (Meyer et al., 2005). Each Polymerase Chain Reaction (PCR) contained 13 μL total volume: 2.5 μL of 10× PCR Rxn Buffer, 3 mM MgCl2, 0.2 μM of dNTPs, 0.2 M of forward primer, 0.2 M of reverse primer, 1 unit of Taq Platinum DNA Polymerase 5000 U Invitrogen, and 20 ng of DNA. Conditions for PCR amplification using the first pair of primers were the following: initial denaturation at 95°C for 2 min; 40 cycles of 95°C for 2 min, annealing at 46°C for 1 min, and extension at 72°C for 1 min; final extension at 72°C for 5 min. Conditions for PCR amplification using the second pair of primers were the following: initial denaturation at 94°C for 3 min; 35 cycles of 94°C for 45 s, annealing at 47°C for 30 s, and extension at 72°C for 1.5 min; final extension at 72°C for 10 min. The results were confirmed through an agarose gel electrophoresis. The amplifications were then treated with ExoSAP before being sent to Macrogen Inc. for sanger sequencing.

### 2.2 Gene tree and genetic diversity

A phylogenetic gene tree was inferred with the COI fragments amplified with the Jerry and Pat primers—GenBank accession numbers: MW041883–MW0441891. The sequences obtained with the primers dgLCO1490 and dgHCO2198 correspond to the COI fragment traditionally used in DNA barcoding efforts (Waugh, 2007) and are available as references for further comparisons—GenBank accession numbers: MW0441892–MW0441898. We did not include these sequences in our gene tree because there are no publicly available sequences in GenBank or elsewhere obtained with these primers for any of the species of the *G. scutellatus* complex. Our newly generated DNA sequences were aligned with previously published sequences (Garcia et al., 2019; Mapondera et al., 2012) and with other sequences available in GenBank for the *G. scutellatus* species complex using the Geneious Alignment tool in Geneious Prime 2020.0.3 (https://www.geneious.com/). This alignment tool is versatile because it can detect, and automatically transform, the direction of the sequences. The final matrix consisted of 152 sequences, including two sequences of *Oxyops* sp. as outgroups, and it had a length of 420 bp. We used IQ‐Tree 1.6.12 (Nguyen et al., 2015) to determine in the same analysis the best‐fitting evolutionary model, the best maximum likelihood tree, and three measures of nodal support. With the option ModelFinder (Kalyaanamoorthy et al., 2017), we selected the best‐fitting model of codon substitution (MGK + F3X4 + G4), because codon substitution models are more realistic than other types of substitution models (i.e., nucleotide, amino acid) in protein‐coding sequences (Gil et al., 2013; Seo & Kishino, 2009). Nodal support was estimated with the SH‐like approximate likelihood ratio test (SH‐aLRT), using 1000 bootstrap replicates (Guindon et al., 2010), the aBayes test, which is a Bayesian‐like transformation of aLRT (Anisimova et al., 2011), and the ultrafast bootstrap approximation, using 1000 replicates (Hoang et al., 2018; Minh et al., 2013). For the six *Eucalyptus* snout beetle species with most samples (*n* ≥ 10), we calculated haplotype diversity (*H*) and nucleotide diversity (π) with the functions hap.div and nuc.div from the package *pegas* in R v3.6.3 (Paradis, 2010; R Core Team, 2020). *H* is the probability that two randomly drawn DNA sequences from the sample would be different; thus, the values of *H* range from 0 to 1, with 0 indicating that all sequences are identical and 1 corresponding to a highly diverse sample because all sequences are different. π is the average number of differences per nucleotide site, among pairwise comparisons of DNA sequences of the sample; thus, larger values indicate greater nucleotide diversity (Nei, 1987).

### 2.3 Occurrence data mining

After confirming the records in Ecuador as *G. platensis*, we generated a database of 347 occurrence records of this species available in the Global Biodiversity Information Facility (GBIF.org, 2022; www.gbif.org; https://doi.org/10.15468/dl.8mwpht). The occurrence dataset included few records from the native range of *G. platensis* in Australia (*n* = 9 records with coordinates; GBIF.org, 2022; www.gbif.org; https://doi.org/10.15468/dl.26tsqw). Therefore, we focused on characterizing the potential distribution of the species only using records from the invaded range in South America. All records corresponded to iNaturalist Research Grade observations (i.e., observation records that have a photograph, date, and coordinates and are verified with agreement by the iNaturalist community). Occurrence records were then verified to ensure georeferencing accuracy, first, by excluding localities without spatial reference and duplicates, and then, by visual examination mapping using the packages *sp* and *maptools* (Bivand & Lewin‐Koh, 2022) in R v3.6.3 (R Core Team, 2020). Finally, occurrences were rarefied to an extent of >5 km spatial distance from each other using the R package *raster* (Hijmans, 2023). The 5 km spatial buffer matched the resolution of the environmental data (see below), and thinning occurrence datasets helps reduce bias when modeling the distribution of invasive species (Elith et al., 2010). The final species occurrence dataset of *G. platensis* included 52 unique localities that were used for modeling. Data are available for download in Zenodo (https://doi.org/10.5281/zenodo.7818068).

### 2.4 Predicted distribution of suitable habitat

**FIGURE A1:**
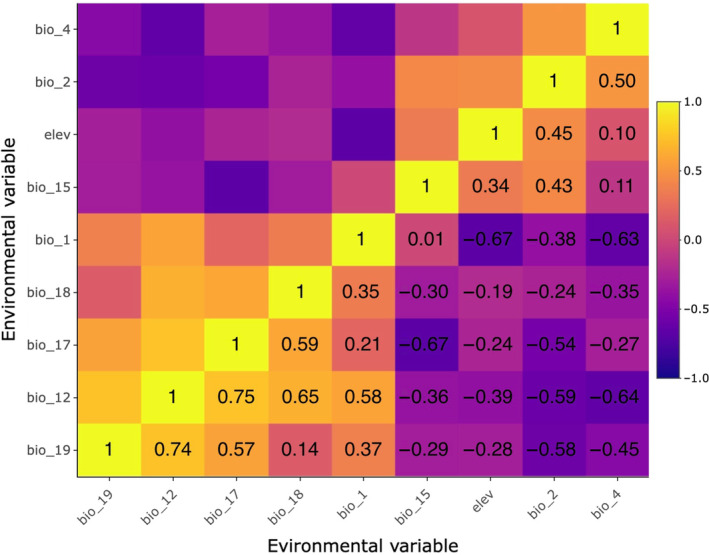
Correlation matrix and heatmap of environmental variables used in niche modeling analysis. Nine WorldClim (www.worldclim.org) environmental variables were retained based on a Pearson correlation coefficient > 0.8. Temperature variables included Annual Mean (bio1), Mean Diurnal Range (bio2), and Seasonality (bio4). Precipitation variables included annual (bio12), seasonality (bio15), driest quarter (bio17), warmest quarter (bio18), and coldest quarter (bio19). Elevation was also retained.

We used presence only data analyzed under two alternative distribution modeling approaches. First, we modeled habitat suitability of *G. platensis* using an ensemble distribution modeling method. Second, we implemented a maximum entropy approach to develop ecological niche models (ENMs). Our goal was to evaluate the predicted distribution of *G. platensis* in its introduced range throughout South America, with a focus on Ecuador. We used climate data from 19 WorldClim variables summarizing temperature and precipitation features (Fick & Hijmans, 2017) and elevation. Environmental data were trimmed to the regional extent of South America using the R package *raster* (Hijmans, 2023). To reduce collinearity among WorldClim variables, we conducted a Pearson correlation using a threshold of 0.8 (Petitpierre et al., 2017; Soto‐Centeno & Simmons, 2022). The final set of uncorrelated environmental variables included: Annual Mean Temperature (bio1), Mean Diurnal Range (bio2), Temperature Seasonality (bio4), Annual Precipitation (bio12), Precipitation Seasonality (bio15), Precipitation of Driest Quarter (bio17), Precipitation of Warmest Quarter (bio18), Precipitation of Coldest Quarter (bio19), and elevation (Figure A1). The same environmental variables were used for all ENM analyses. The choice of environmental background can influence the predictive ability in ENM (Elith et al., 2010). Therefore, we created a background extent to calibrate the ENM by generating a buffer of 500 km around each observed locality of *G. platensis*, and sampling 10,000 random points within that environmental extent. Final models were then projected onto the regional extent of South America.

Ensemble ENMs were produced using the R package SSDM v0.2.8 and combined four algorithms, generalized linear model (GLM), Maxent, random forest (RF), and support vector machine (SVM; Schmitt et al., 2017). Ensemble distribution modeling approaches have been used to study the distribution of invasive species (Roura‐Pascual et al., 2009). This approach accounts for variability across modeling algorithms to provide a measure of central tendency where consensus areas of habitat suitability exist (Araujo & New, 2007; Roura‐Pascual et al., 2009). We set a combination of default and custom parameters for each modeling algorithm in the ensemble approach (see ?ensemble_modeling in SSDM v0.2.8). For GLM, we set the default parameters of *epsilon* = 1e‐08 and *maxit* = 500; for Maxent, we estimated custom parameters using the R package *ENMeval2.0* (Kass et al., 2021; details below); for RF, we set *ntree* = 5000 with *nodesize* = 1; and for SVM, we set a default *epsilon* = 1e‐08, *cross* = 5 (i.e., *k*‐fold cross‐validation = 5), *kernel* = radial, and default *gamma* = 1/(length(data) − 1).

Recent studies show that ensemble modeling performs well, but not always consistently better than single model approaches that could be better parameterized (Hao et al., 2020). Thus, as a complement, we also produced an ENM based on a single algorithm using Maxent v3.4.1. This method is widely used and shows high predictive performance compared with other modeling methods (Elith et al., 2006; Phillips et al., 2006). Furthermore, R packages have been developed to explore custom‐tuning parameters that best fit the data (Kass et al., 2021). The Maxent model was fine tuned to maximize its predictive performance. Specifically, species localities were randomly partitioned into 75% training and 25% testing datasets, and model calibration followed a cross‐validation approach with *k* = 5 to reduce overfitting. We evaluated a range of regularization values from 1 to 5 and combinations of up to four feature classes (i.e., linear, quadratic, hinge, linear‐quadratic, linear‐quadratic‐hinge, and linear‐quadratic‐hinge‐product) in the R package *ENMeval2.0* (Kass et al., 2021). The best tuning parameters for Maxent modeling were then selected using Akaike information criterion (AIC; Table A1). Maxent uses regularization to reduce model complexity and included variables contribute differentially to the final model (Phillips & Dudík, 2008). The final model was calibrated using the background extent and the best tuning parameters (i.e., fc = LQHP and rm = 2) and was projected on South America.

Model performance for ensemble and Maxent models was assessed using the area under the receiving operating characteristic curve (AUC). AUC is a threshold‐independent measure that varies from 0 to 1, where a score of 1 represents perfect discrimination and a score of 0.5 represents a model no better than random (Peterson et al., 2011). We considered an AUC score greater than 0.7 to represent good model predictions (Peterson et al., 2011). Given that AUC has been deemed unreliable for estimating performance of presence‐background models (e.g., Lobo et al., 2008) we separately calculated the Boyce Index (BI) in Maxent models to assess model prediction in the R package *ecospat* (Di Cola et al., 2017; Hirzel et al., 2006). The BI uses a Spearman rank correlation coefficient, which varies from −1 to 1 (Hirzel et al., 2006). A positive BI value approaching one indicates that model predictions are consistent with the evaluation dataset, zero indicates random performance, and negative values indicate a poor match with the evaluation dataset (Hirzel et al., 2006).

Because *G. platensis* is invasive in South America, all final projected models implemented a lowest presence threshold of 95% (LPT95, equivalent to the Minimum Training Presence threshold) (Soto‐Centeno & Steadman, 2015). Under this rule, prediction pixels with equal or higher values than the LPT95 were scored as suitable conditions where *G. platensis* could sustain viable populations in the introduced range. We chose LPT95 to provide a conservative prediction where model datasets contained at least 95% of locality points within suitable habitat (i.e., a theoretical expectation of 5% omission rate of the training data; Pearson et al., 2007). This threshold also helped us determine whether our ENMs allowed enough sensitivity to predict the observed localities and examine novel areas of environmental suitability where *G. platensis* could establish populations in South America.

### 2.5 Characterization of climate envelopes

We examined the range of conditions where *G. platensis* is found throughout South America (i.e., their “climate envelope”; Hijmans & Graham, 2006). These ranges were compared to the conditions where the species is found in Ecuador. This helped us define whether conditions in Ecuador differ from those in the rest of the continent. Climate envelopes were constructed using data from the top four environmental variables with highest contributions (i.e., >8%) in the ensemble models and elevation. In order of importance, the variables included annual mean temperature (bio 1), temperature seasonality (bio 4), precipitation seasonality (bio 15), elevation, and precipitation of the coldest quarter (bio 19). Elevation had a 9.5% contribution to the model, but we used it here to broadly discuss areas of predicted suitable habitat of *G. platensis*. From these data, we determined the climate and elevation profiles for all localities where *G. platensis* was documented by extracting environmental information across all localities and directly comparing the range of conditions individually in Ecuador vs. the rest of South America. These data were not normally distributed; thus, Wilcoxon rank sum tests followed by a Bonferroni correction for multiple comparisons were used to examine the differences between conditions in Ecuador vs. the rest of South America. This framework allowed us to better understand the variation in environmental niche occupancy in the introduced range of *G. platensis* across the continent.

### 2.6 Association of habitat suitability of *Gonipterus* and *Eucalyptus* species richness

*Eucalyptus* trees are the primary host for *G. platensis* worldwide, but their host specificity or susceptibility are poorly understood, especially in tropical areas (Oliveira et al., 2022). To better understand the drivers of distribution of *G. platensis* in South America, we examined the association of *G. platensis* with the host *Eucalyptus* trees. We obtained 15,093 observation records of seven species of *Eucalyptus* trees that have been introduced in South America (see Table A2 for details about the original data). Occurrence records of *Eucalyptus* were verified following the same scheme as above and rarefied to a 5 km spatial resolution. The final species occurrence dataset of *Eucalyptus* included 264 unique localities of five species *E. camaldulensis* (*n* = 16), *E. globulus* (*n* = 162), *E. grandis* (*n* = 36), *E. urophylla* (*n* = 16), and *E. viminalis* (*n* = 34). Data are available for download in Zenodo (https://doi.org/10.5281/zenodo.7818068).

We modeled five species of *Eucalyptus* trees in South America using a community pSSDM approach in the R package SSDM v0.2.8 (Schmitt et al., 2017). This approach uses sum probabilities of habitat suitability based on ensemble models to produce a single map of species richness. The modeling algorithms used for pSSDM were GLM, Maxent, RF, and SVM under default parameters. The resulting stacked community model map of species richness of *Eucalyptus* across South America was evaluated using sensitivity and individual model AUC values. We then used a linear regression analysis to examine the relationship of *Eucalyptus* species richness with the predicted habitat suitability of *G. platensis*.

## 3 RESULTS

### 3.1 Gene tree, molecular identification, and genetic diversity

**FIGURE 2:**
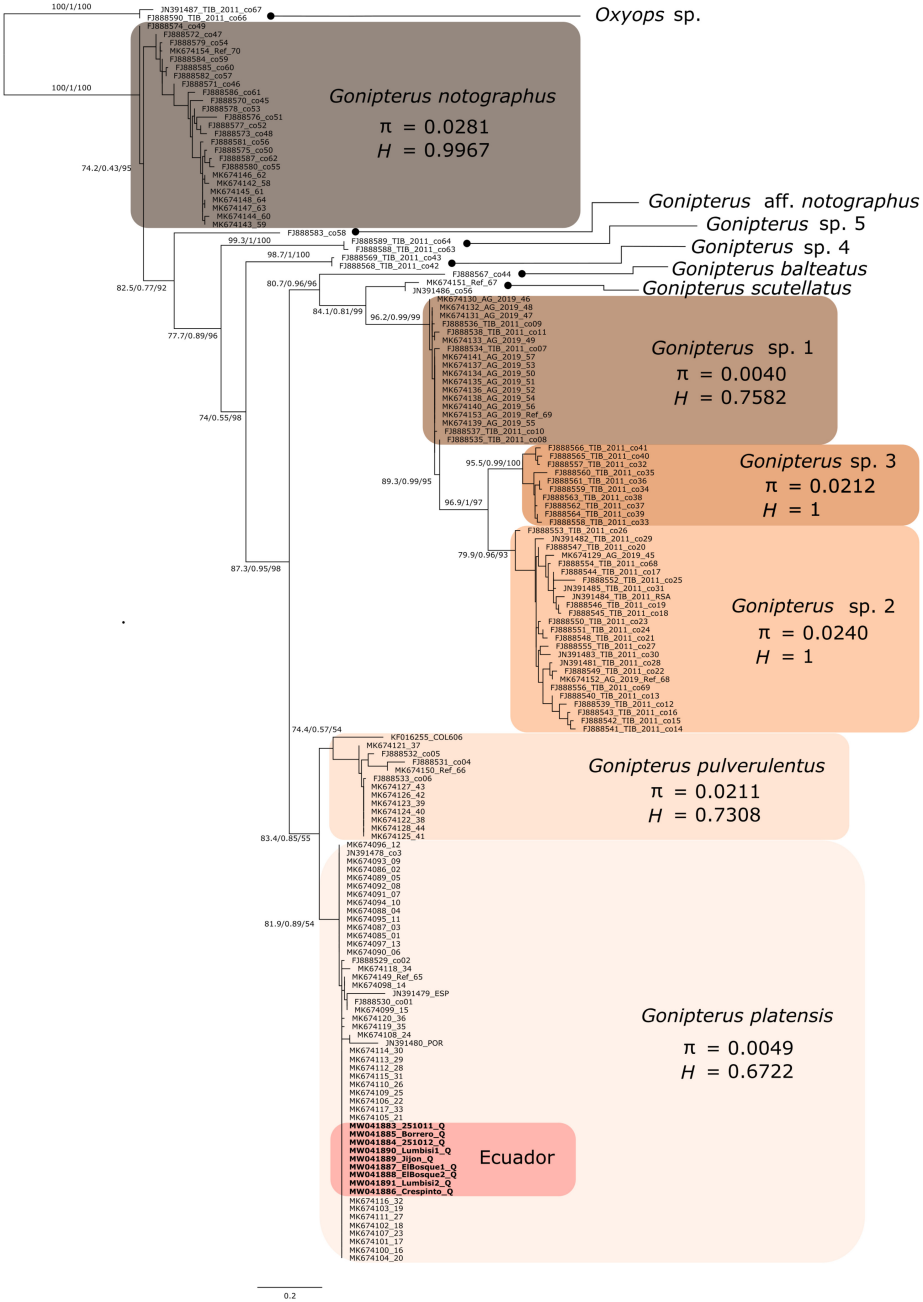
Maximum likelihood tree of a fragment of the COI gene of species of the *Gonipterus scutellatus* complex. Numbers at the nodes indicate support values from the SH‐like approximate likelihood ratio test (SH‐aLRT), the Bayesian‐like transformation of aLRT (aBayes test), and the ultrafast bootstrap method. Terminal labels indicate the GenBank accessions, followed by sample codes. Clade classification follows Mapondera et al. (2012). Values of nucleotide (π) and haplotype (*H*) diversity for each of the six species examined are indicated below the species names. *Gonipterus* sp. 1, 2, 3, 4, and 5 are separate (albeit undescribed) species based on morphological and molecular evidence (Garcia et al., 2019; Mapondera et al., 2012). Most samples were collected in mainland Australia and Tasmania, except for our samples (in bold) and samples: JN391484_TIB_2011_RSA, JN391479_ESP, and JN391480_POR, which were collected in South Africa, Spain and Portugal, respectively. The scale bar represents the mean number of nucleotide substitutions per site.

The gene tree of the mitochondrial COI gene of species of the *G. scutellatus* complex (Figure 2) showed that the Ecuadorian samples are nested within the clade of *G. platensis*. The Ecuadorian samples were identical among them and showed no variation with most of the other *G. platensis* in the clade. In fact, across the dataset, *G. platensis* was one of the least genetically diverse species (see nucleotide diversities, π, in Figure 2), despite being the one with the most representation of DNA sequences. On the contrary, *G. notographus* and *G*. sp. 2 were the most genetically diverse species. Note that one sample, FJ888583_co58, here designated as *G*. aff. *notographus*, was placed outside the *G. notographus* clade.

### 3.2 Predicted distribution, climate envelope, and host plant association

**FIGURE 3:**
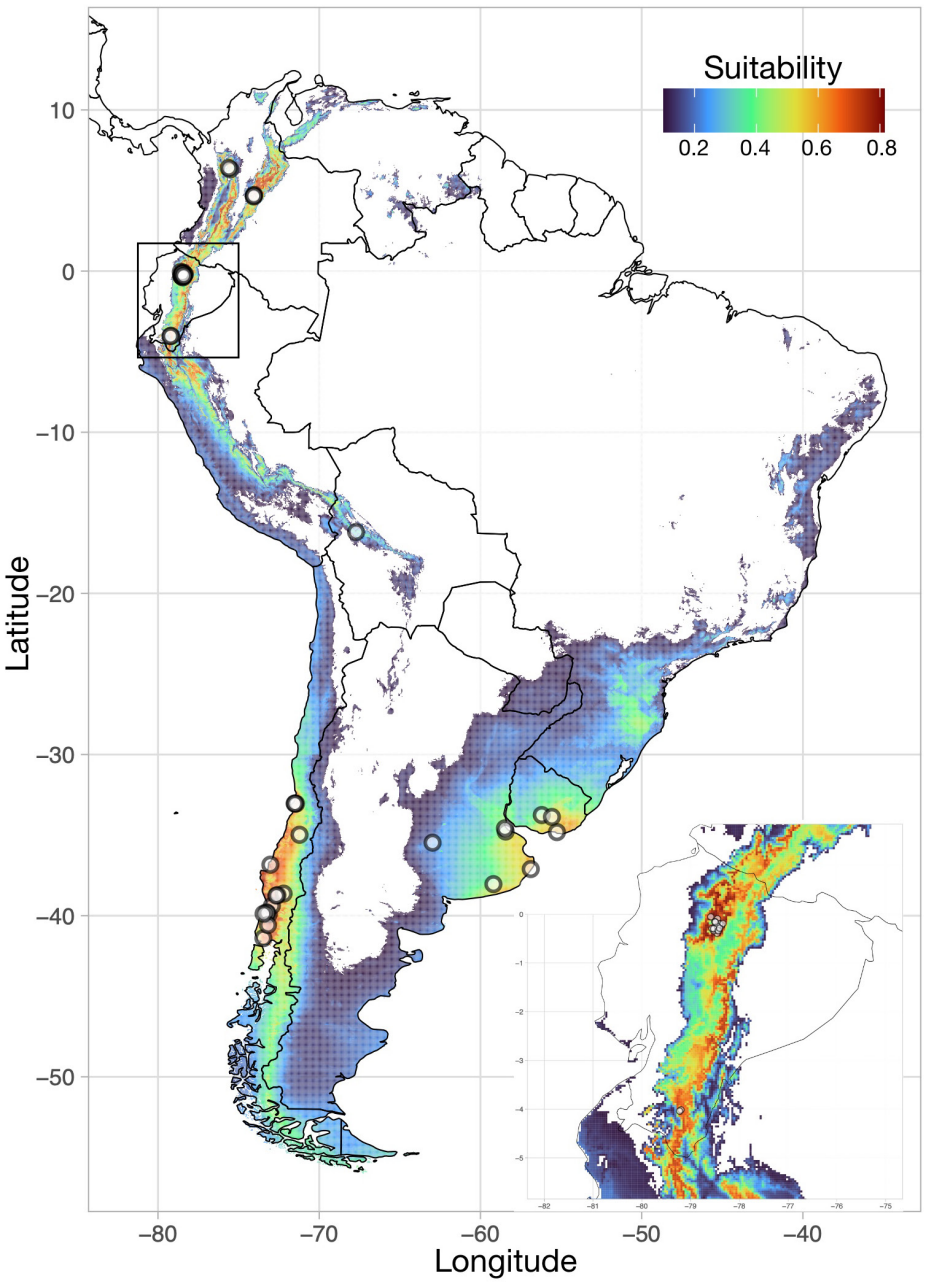
Predicted distribution of *Gonipterus platensis* in its introduced range of South America. The inset shows a focus on Ecuador to emphasize habitat suitability detail. The ensemble model was calibrated to the environmental conditions of the introduced range in South America. Dots represent observations used for modeling. Warmer colors of predicted distribution (i.e., red) indicate higher environmental suitability. Niche model predictions based on a single algorithm, Maxent, are included in the Figure A2.

**FIGURE 4:**
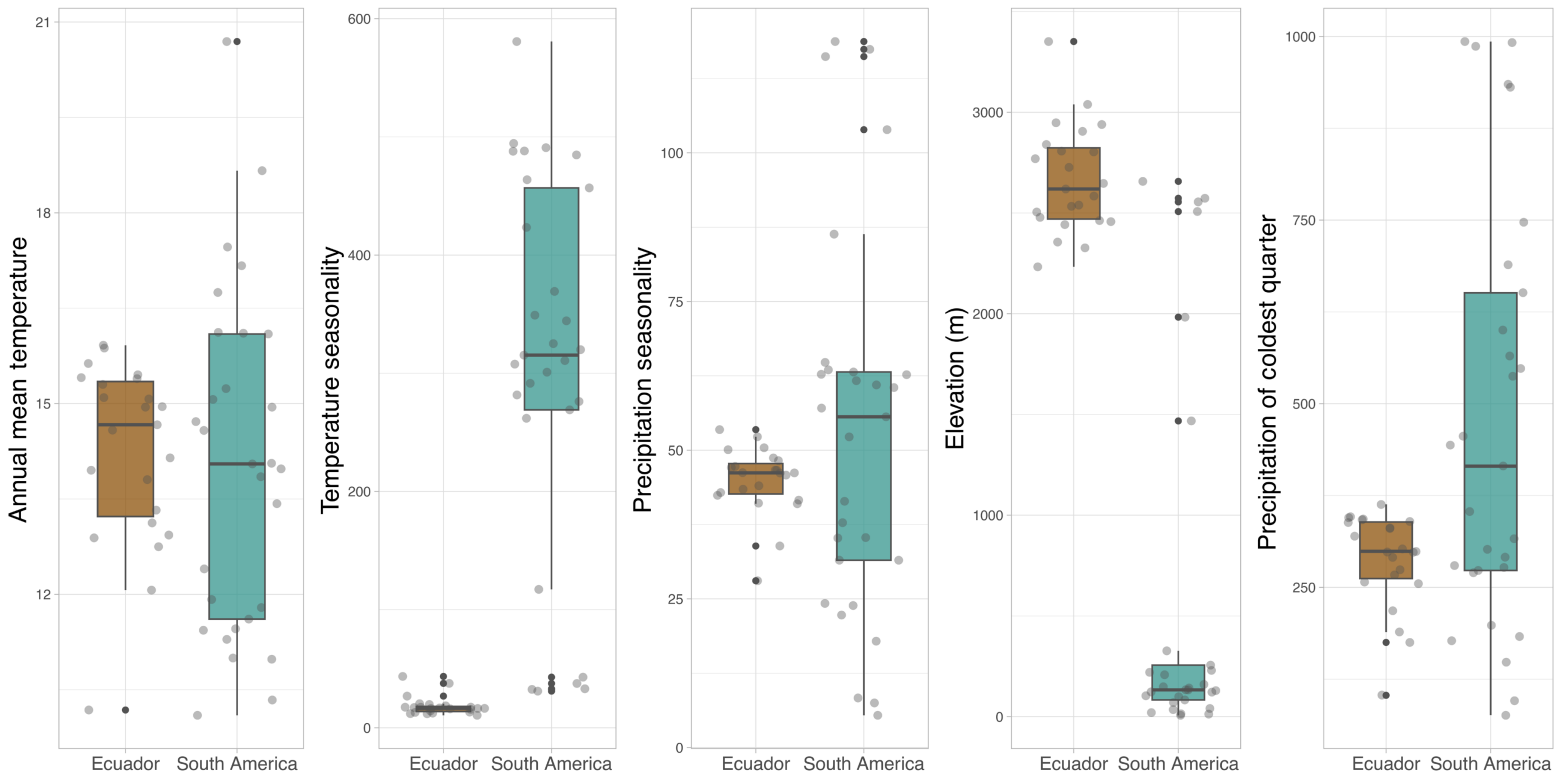
Boxplots showing the distribution for environmental features characterizing the introduced ranges of *Gonipterus platensis*. Brown = Ecuador and teal = South America. Median: heavy line; 25% and 75% quartiles: edges of box; 5% and 95% quartiles: whiskers; observed values included as points to help visualize density.

**FIGURE A2:**
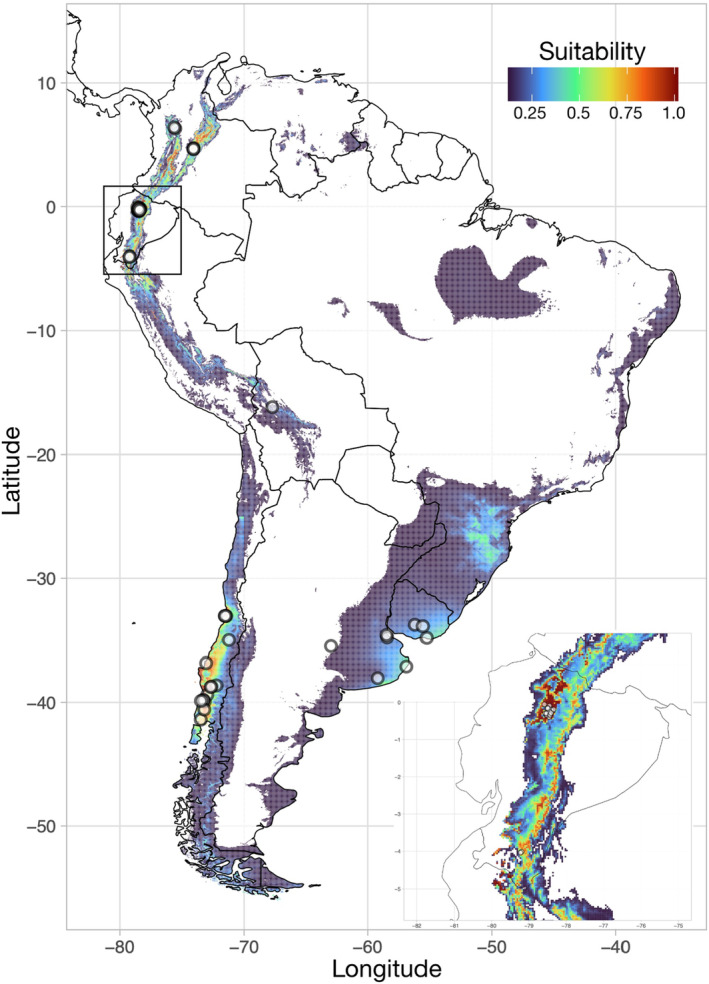
Maxent model (i.e., single algorithm) predicted distribution of *Gonipterus platensis* in its introduced range of South America. The inset shows a focus on Ecuador to emphasize habitat suitability detail. The model was calibrated to the local environmental conditions of the introduced range in South America (see main text for model parameter details). Dots represent observations used for modeling. Warmer colors of predicted distribution (i.e., red) indicate higher environmental suitability.

As an invasive species, the distribution of *G. platensis* in South America could be driven by an expansion into novel environments. Thus, our ENM approach focused on evaluating areas of potentially suitable climate that the species could invade. The ensemble mode of *G. platensis* had good performance with an overall AUC value of 0.94 and had a true positive fraction of 100% (Figure 3). Similarly, each individual algorithm within the ensemble model performed well (GLM AUC = 0.83, Maxent AUC = 0.98, RF AUC = 0.96, and SVM AUC = 0.95). The standalone parameterized Maxent model had good performance and obtained a true positive fraction of 98% across all known unique localities of the invaded range (AUC = 0.956, BI = 0.883; Figure A2). Ensemble and standalone Maxent models performed similarly in terms of statistics and geographic projections; we discuss our results based on the ensemble model because of the higher true positive fraction. Notably, this ENM predicted highly suitable climates for *G. platensis* in the Andes of Colombia, Ecuador, and Northern Peru. The coastal portion Chile, from Santiago to Puerto Montt, also showed high suitability, whereas areas at the southeast of Brazil and Uruguay, east Argentina and the Andes of Bolivia showed intermediate suitability. In Ecuador, we found three areas of high suitability in the northern (where we collected the specimens analyzed in this study), central and southern parts of the Ecuadorian Andes. Comparison of climate envelopes of the four top contributing climatic factors and elevation for Ecuador and the rest of South America highlighted some variation in the occupied niches (Figure 4 and Table 1). For example, the range of *G. platensis* in Ecuador revealed significant climate envelope differences related to seasonality and elevation (Figure 4 and Table 1). Across South America, these beetles occupy a wide breadth of climatic conditions and elevations. They exist in elevational ranges spanning low and high altitudes, from 7 to 3351 m above sea level (a.s.l.). In Ecuador, *G. platensis* occupies the highest elevational range with a mean of ca. 2600 (2233–3351) m a.s.l. (Figure 4). The lowest elevation ranges were found in Argentina, Chile, and Uruguay, generally found from 7 to 327 m a.s.l.

**TABLE 1:** Environmental comparison of climate envelopes for *Gonipterus platensis* in South America.

Locality	*n*	Bio 1	Bio 4	Bio 15	Elevation	Bio 19
Ecuador	23	14.2 (10–16)	18.3 (11–44)	44.9 (28–53)	2666 (2233–3351)	288 (363–993)
South America	29	14.1 (10–21)	310 (31–581)	52.8 (5–119)	566 (7–2657)	473 (76–993)
	*p*	>.5	<.005*	>.5	<.005*	>.1

*Note*: All values represent means (range) for the following environmental features: Annual mean temperature (bio 1), Temperature seasonality (st.dev. × 100; bio 4), precipitation seasonality (bio 15), elevation (in m a.s.l.), and precipitation of the coldest quarter (bio 19). *p*‐values adjusted using a Bonferroni correction; values with an asterisk (*) indicate statistical significance at the .05 level.

**FIGURE A3:**
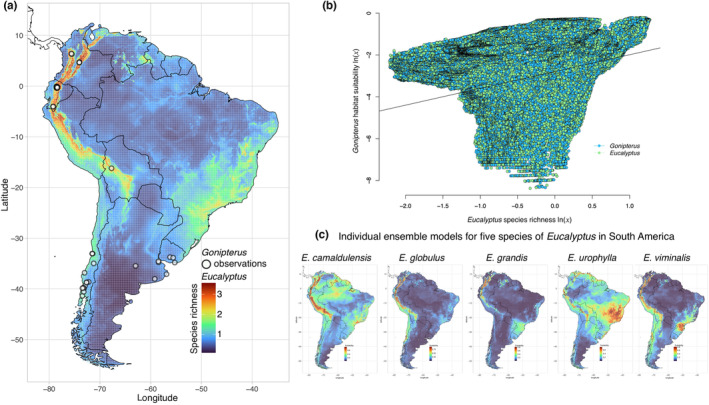
Summary of results of the relationship of *Eucalyptus* species richness with habitat suitability of *Gonipterus platensis*. (a) Stacked species distribution model (pSSDM) showing species richness of five species of *Eucalyptus* trees in South America. Dots represent the observation localities of *G. platensis* overlaid over the *Eucalyptus* species richness map to aid in visualization. (b) Linear regression of the relationship of *Eucalyptus* species richness and the predicted habitat suitability of *G. platensis* (*F* = 1.214e + 05, *DF* = 1 and 886,703, *p* < 2.2e‐16, *R* ^2^ = .1204). (c) pSSDM was built based on individual *Eucalyptus* species ensemble models using four modeling algorithms [i.e., generalized linear model (GLM), maxent, random forest (RF), and support vector machine (SVM)]. Performance results from each individual species model: *Eucalyptus camaldulensis* average AUC = 0.76, *Eucalyptus globulus* average AUC = 0.91, *Eucalyptus grandis* average AUC = 0.99, *Eucalyptus urophylla* average AUC = 0.78, and *Eucalyptus viminalis* average AUC = 0.95.

*Eucalyptus* trees have been broadly introduced throughout South America (FAO, 1981). Our community pSSDM model for five species of *Eucalyptus* performed well, with an assemblage sensitivity rate (i.e., proportion of true positives) of 0.96, and good individual model AUC values (GLM AUC = 0.83, Maxent AUC = 0.82, RF AUC = 0.87, and SVM AUC = 0.85). This model showed the Andes from Venezuela to Bolivia hosting the highest species richness of *Eucalyptus* and closely matching the distribution of *G. platnensis* in that region (Figure A3a). The lowest predicted species richness of *Eucalyptus* was observed in eastern Argentina and Uruguay. The predicted habitat suitability of *G. platensis* shows a significant positive relationship with *Eucalyptus* species richness (Figure A3b; *F* = 1.214e+05, *DF* = 1 and 886,703, *p* < 2.2e‐16). However, the data showed a weak fit to the regression model (*R* ^2^ = .1204).

## 4 DISCUSSION

We aimed to document the introduction of the *Eucalyptus* snout beetle in Ecuador, determine its taxonomic placement, and model potentially suitable habitat throughout South America. With this framework, we provide the first genetic evidence for the presence of *G. platensis* in Ecuador. Furthermore, our ecological niche model analysis suggests areas of suitable habitat throughout a broad range of climatic and elevational regimes that may have played a role in the establishment of populations of *Eucalyptus* snout beetles in South America and could promote its future expansion into new areas.

The gene tree highlighted large genetic variation across the *G. scutellatus* species complex. However, most of the samples analyzed in this study came from Australia and Tasmania, with few samples coming from Portugal, Spain, and South Africa (Mapondera et al., 2012), and only the nine samples we collected coming from South America. DNA barcoding data (mitochondrial COI sequences) clearly resolved the identity of the beetles found in Ecuador as *G. platensis*, which has also been documented in other South American countries (i.e., Argentina, Brazil, Colombia, Chile, and Uruguay) (Garcia et al., 2019; Mapondera et al., 2012; Schröder et al., 2020). Although the ultrafast bootstrap value is low (55%) for the *G. platensis* clade (Figure 2), analyses of similar datasets with less terminals have recovered higher values for the *G. platensis* clade: A Bayesian posterior probability of 0.96 in Mapondera et al. (2012), and a bootstrap value of 88% in Garcia et al. (2019). The differences among these support values could be related to the use of different models of evolution. We used a codon substitution model, while Garcia et al. (2019) and Mapondera et al. (2012) used nucleotide substitution models. The genetic diversity of *G. platensis* was one of the lowest among the species analyzed in this study (see values of π for each clade in Figure 2), which coincides with the results found by Mapondera et al. (2012) for this species in Western Australia. In fact, the Ecuadorian samples share the same haplotype with individuals from Tasmania (Garcia et al., 2019), which suggests that the Ecuadorian population could have originated from a Tasmanian source. Notwithstanding, analyses including more loci (perhaps genomes) from *G. platensis* from Colombia, other South American countries and from its native range are needed to thoroughly examine whether the Ecuadorian samples were a product of a direct introduction from the native range or if they were secondarily introduced from another South American country. An introduction from Colombia seems plausible given geographical proximity and the continuity of suitable climatic habitat predicted by our model (Figure 3). Moreover, our gene tree suggested that there are at least three different geographical origins of the *G. platensis* specimens found outside Australia. Indeed, two different sequences of this species from Spain and Portugal (JN391479_ESP and JN391480_POR) are the most different compared with the sequences of South America and Tasmania, as evidenced by their long branches in the gene tree (see *G. platensis* clade in Figure 2). Additional sampling (i.e., individuals and loci) and genetic analysis are necessary from *Eucalyptus* snout beetle populations from Southeastern Australia and from other South American countries to truly pinpoint the origin of invasive populations.

Two main challenges have been identified when producing ecological niche models (ENM) for invasive species (Lake et al., 2020). First, invasive species are often in disequilibrium with the novel environment they occupy, and second, generating ENM projections from native to invaded ranges may be problematic for correlative model approaches (Elith et al., 2010). We followed an ensemble ENM approach at the species level, including all unique localities of *G. platensis* in South America available in GBIF. This approach is justifiable because very few records (with coordinates) were available in open‐source databases from the native range of Australia (*n* = 9). Indeed, little research has been carried out on *Eucalyptus* snout beetles in their native range, probably because in Australia they are only minor pests (Clarke et al., 1998). Such a low number of occurrences would bias ENM predictions, particularly when projected from native ranges into novel environments where the species was introduced. Furthermore, using only the native range to estimate the potential suitable habitat of a species may result in misrepresentation of predictions, particularly if the colonization into the invaded range is characterized by niche expansion (Broennimann et al., 2007). To mitigate the spatial data bias, we rarefied records and used a background extent that included only potentially reachable areas (following Elith et al., 2010). This choice of background would reduce the degree of model extrapolation (Elith et al., 2010). Despite the uneven sampling, our approach allowed us to produce estimates of the areas where *G. platensis* occurs, but also assess regions of suitable habitat where these beetles could potentially be found or invade (Figure 3).

The ENM predictions attained a high true positive fraction for the currently known observations (Figure 3 and Figure A2). Notably, the ensemble model predicted occurrences in western Buenos Aires province of Argentina and the Andes of Bolivia, with intermediate predicted distribution values. Particularly, the inland locality of Argentina was characterized by a narrow range of high temperatures (i.e., little variation in temperature seasonality) and generally dry conditions (i.e., lowest precipitation of coldest quarter) that were unique compared with all other occurrences. Despite the lack of observation records from GBIF, model predictions showed that suitable habitat for *G. platensis* exists in southeastern Brazil in the city of Curitiba, PR, where the beetle was first documented as invasive for the country (Figure 3; op. cit. *G. scutellatus* in Freitas, 1979). This region of suitability in Brazil spans from the state of Espírito Santo, south through Rio Grande do Sul, and west into Mato Grosso do Sul, in which *G. platensis* is widely recognized as a pest of *Eucalyptus* forests (de Souza, 2016; Wilcken & Oliveira, 2015).

In the northwestern parts of South America, ensemble ENM predicts suitable habitat across the Andes from Colombia to Bolivia at high elevations (Figure 3). In Ecuador, *G. platensis* was predicted to have significantly higher elevations than elsewhere in the continent (Figure 4 and Table 1), with highly suitable habitat existing in and around the cities of Quito, Ambato, and Cuenca and with at least moderately suitable habitat occurring all along the Ecuadorian Andes (Figure 3). Suitable habitat at high elevations was also predicted throughout Perú, which may represent potential areas for invasion (Figure 3). In the southern countries, the model resulted in high to intermediate values of suitability at lower elevations in Chile, Argentina, and Uruguay, respectively (Figure 3). Examination of the climate envelopes revealed that *G. platensis* in Ecuador occupies significantly less seasonal and drier conditions than in other parts of the invaded range at similar elevations (Figure 4 and Table 1). Nonetheless, when considering the entire distribution, this beetle occurs across a wide elevational range and a variety of suitable environmental conditions where it could potentially sustain long‐term populations.

We found a positive association between areas of high species richness of *Eucalyptus* trees and areas of high environmental suitability for *G. platensis* (Figure A3). Areas along the Andes from Venezuela to Bolivia contain the highest species richness for *Eucalyptus*. Qualitatively, these areas are highly congruent with the predicted distribution of *G. platensis* and emphasize the potential presence or invasive potential of this beetle in southern Colombia, Peru, and Bolivia. This pattern is also apparent across eastern Brazil, where despite the paucity of records of *G. platensis* the breadth of *Eucalyptus* distribution could help the spread of this pest. A higher density of records of *G. platensis* exists in Argentina, Chile, and Uruguay, which are countries of low *Eucalyptus* species richness. Additional studies examining the interactions of *G. platensis* with its host plants could shed light into the potential spread of this beetle in South America.

Two sources of error could render our ENM predictions somewhat exploratory. First, sampling bias likely excluded areas occupied by *G. platensis* but not included in GBIF or not yet documented across South America. Second, even though all records included in our model were iNaturalist research grade observations, some could have been misidentified due to the difficulty of distinguishing between the cryptic species of the *G. scutellatus* complex. Note, however, that the only other *Eucalyptus* snout beetle species registered in South America is *G. pulverulentus*, with, apparently, a limited distribution in Uruguay, according to Schröder et al. (2020), but also in Argentina and Brazil, according to Mapondera et al. (2012). *Gonipterus platensis* is, in contrast, the most widely distributed of the species in the complex, occurring in eastern and western South America (Mapondera et al., 2012). In any case, our ENM results combined with climate envelope analysis highlight the potential of *G. platensis* to occupy a broad range of environmental conditions, which may allow it to become highly invasive in different parts of South America.

Finally, we make a call to study the ecological and economic impacts of the invasion of the *Eucalyptus* snout beetle and its hosts (e.g., *Eucalyptus globulus*) in South America, particularly in Colombia and Ecuador. In northern South America little research has been conducted on forestry of *Eucalyptus* species, whereas in Brazil and Chile, forestry research is quite active. In the particular case of Ecuador, *Eucalyptus* trees were planted in the late 19th century, mainly in volcanic soils, known as “cangagua,” in the inter‐Andean valleys (FAO, 1981). These valleys have had human influence for millennia (Bush et al., 2022; Young, 2009), so perhaps these degraded environments have lost natural enemies that could exploit these insects as new hosts. We thus recommend extensive sampling of *Eucalyptus* snout beetles and potential controllers in Ecuador to test this hypothesis. Also, as far as we know and according to records in GBIF, *G. platensis* remains concentrated in a couple of areas of the Ecuadorian Andes (around the city of Quito in the North and in the city of Loja in the South, Figure A1), but our model predicts the whole range as climatically suitable for invasion. In fact, climatic suitability expands across the border to the Peruvian Andes, within the area of distribution of *Eucalyptus* spp. plantations (Castillo Vera et al., 2019; Luzar, 2007), where no reports of the insects have been made so far. All this highlights the urgency of conducting more studies on the invasion of this pest to design effective control and/or prevention measures.

## AUTHOR CONTRIBUTIONS

**Verónica Crespo‐Pérez:** Conceptualization (lead); data curation (equal); project administration (lead); writing – original draft (lead). **J. Angel Soto‐Centeno:** Conceptualization (supporting); data curation (equal); formal analysis (equal); methodology (equal); writing – original draft (supporting). **C. Miguel Pinto:** Conceptualization (supporting); formal analysis (equal); methodology (equal); writing – original draft (supporting). **Ana Avilés:** Data curation (supporting); writing – review and editing (equal). **Washington Pruna:** Data curation (supporting); writing – review and editing (equal). **Claudia Terán:** Methodology (supporting); writing – review and editing (equal). **Álvaro Barragán:** Conceptualization (lead); data curation (equal); project administration (supporting); writing – review and editing (equal).

## FUNDING INFORMATION

This work was supported by PUCE's Research Directorate to projects: “Presencia del gorgojo del eucalipto en Ecuador y riesgo potencial para América del Sur” and M13434. Work by JAS‐C was partly supported by a National Science Foundation grant DEB:2135257.

## CONFLICT OF INTEREST STATEMENT

The authors have declared that no competing interests exist.

### OPEN RESEARCH BADGES

This article has earned an Open Data badge for making publicly available the digitally‐shareable data necessary to reproduce the reported results. The data is available at https://doi.org/10.5281/zenodo.7823745 and https://doi.org/10.5281/zenodo.7818068.

## Data Availability

DNA sequences: Genbank accession numbers MW041883–MW0441898. Files and scripts used for molecular identification are publicly available at Zenodo: https://doi.org/10.5281/zenodo.7823745. Records of *Gonipterus platensis* used for our models and R scripts for Ecological Niche Modeling are publicly available at Zenodo: https://doi.org/10.5281/zenodo.7818068.

## APPENDIX 1

**TABLE A1:** Parameter tuning results from ENMeval (Kass et al., 2021) for *Gonipterus platensis* throughout South America.

Fc	Rm	AUC train	AICc	Delta AICc	ncoef
LQHP	2	0.957554808	1048.293297	0	13
LQHP	1	0.961981731	1057.460745	9.167447733	21
LQH	2	0.954491346	1065.190998	16.89770028	13
LQHP	3	0.955649038	1066.165553	17.87225589	12
LQHP	4	0.954991346	1081.24312	32.94982232	10
H	2	0.955970192	1084.208704	35.9154067	15
LQH	1	0.959727885	1086.294277	38.00097914	23
LQH	3	0.950295192	1087.920779	39.62748184	13
H	1	0.960218269	1093.423031	45.12973404	23
LQHP	5	0.954658654	1100.202585	51.90928807	9
H	3	0.952102885	1105.773464	57.48016651	14
H	4	0.948214423	1122.090168	73.79687027	12
LQH	4	0.944683654	1124.393061	76.09976323	16
LQ	1	0.938035577	1129.562191	81.26889392	14
LQH	5	0.939625962	1130.735969	82.44267122	12
H	5	0.946266346	1139.439898	91.14660089	11
LQ	2	0.932877885	1161.072388	112.7790909	13
LQ	3	0.929779808	1190.87759	142.5842929	12
LQ	4	0.924974038	1216.858404	168.565107	12
LQ	5	0.917049038	1231.248262	182.9549645	10
L	1	0.821968269	1344.527095	296.2337974	8
L	2	0.826758654	1346.430869	298.1375719	8
L	3	0.829145192	1353.660729	305.3674317	9
L	5	0.823139423	1357.934525	309.6412272	7
L	4	0.828589423	1358.166874	309.873577	8

Abbreviations: AICc, corrected Akaike Information Criterion; delta.AICc, deviation from the smallest AICc value (set at zero and arranged in ascending order); fc, feature classes (i.e., H, hinge; L, linear; P, product; Q, quadratic); ncoef, number of coefficients; rm, regularization (beta) multiplier.

**TABLE A2:** Number of records and URL of crude data obtained from GBIF (www.gbif.org) used to model *Eucalyptus* spp. distribution in South America.

*Eucalyptus* species	Number of data points	URL
*Eucalyptus camaldulensis*	114	https://doi.org/10.15468/dl.6vmerd
*Eucalyptus dunni*	44	https://doi.org/10.15468/dl.tzttxj
*Eucalyptus globulus*	11,416	https://doi.org/10.15468/dl.pp384n
*Eucalyptus grandis*	1903	https://doi.org/10.15468/dl.8hewnf
*Eucalyptus nitens*	4	https://doi.org/10.15468/dl.nge6xs
*Eucalyptus urophylla*	98	https://doi.org/10.15468/dl.bdp8vu
*Eucalyptus viminalis*	1516	https://doi.org/10.15468/dl.azaqkq
